# Risk factors for outbreaks of COVID‐19 in care homes following hospital discharge: A national cohort analysis

**DOI:** 10.1111/irv.12831

**Published:** 2021-02-06

**Authors:** Chris Emmerson, James P. Adamson, Drew Turner, Mike B. Gravenor, Jane Salmon, Simon Cottrell, Victoria Middleton, Buffy Thomas, Brendan W. Mason, Chris J. Williams

**Affiliations:** ^1^ Public Health Wales Cardiff UK; ^2^ Swansea University Medical School Swansea UK

**Keywords:** care homes, COVID‐19 pandemic, hospital discharge, long‐term care, outbreak, time‐dependent Cox regression

## Abstract

**Background:**

The population of adult residential care homes has been shown to have high morbidity and mortality in relation to COVID‐19.

**Methods:**

We examined 3115 hospital discharges to a national cohort of 1068 adult care homes and subsequent outbreaks of COVID‐19 occurring between 22 February and 27 June 2020. A Cox proportional hazards regression model was used to assess the impact of time‐dependent exposure to hospital discharge on incidence of the first known outbreak, over a window of 7‐21 days after discharge, and adjusted for care home characteristics, including size and type of provision.

**Results:**

A total of 330 homes experienced an outbreak, and 544 homes received a discharge over the study period. Exposure to hospital discharge was not associated with a significant increase in the risk of a new outbreak (hazard ratio 1.15, 95% CI 0.89, 1.47, *P* = .29) after adjusting for care home characteristics. Care home size was the most significant predictor. Hazard ratios (95% CI) in comparison with homes of <10 residents were as follows: 3.40 (1.99, 5.80) for 10‐24 residents; 8.25 (4.93, 13.81) for 25‐49 residents; and 17.35 (9.65, 31.19) for 50+ residents. When stratified for care home size, the outbreak rates were similar for periods when homes were exposed to a hospital discharge, in comparison with periods when homes were unexposed.

**Conclusion:**

Our analyses showed that large homes were at considerably greater risk of outbreaks throughout the epidemic, and after adjusting for care home size, a discharge from hospital was not associated with a significant increase in risk.

## 1 BACKGROUND

Care homes are settings in which resident populations typically live in close proximity. Annually, they experience outbreaks of gastrointestinal and respiratory illnesses, including norovirus and influenza, with associated morbidity and mortality; 70% of acute respiratory infection outbreaks in the UK occurred in care homes in the winter of 2018/19.^1^ Outbreak‐associated infections may be introduced via human sources such as new admissions from home or hospital, via staff or via visitors.

Early evidence from the COVID‐19 pandemic, later further corroborated, was that older people were more severely affected, with a case fatality proportion of 2.3% overall but 8% in those aged 70‐79 and 14.8% in those aged over 80.^2^ An assessment of international evidence from April estimated that in Italy and Spain, over half of reported deaths were in care home residents.^3^

Preliminary studies from April in England found extensive spread amongst staff and residents in homes reporting incidents, and wide variation in symptom profiles.^4^ A Scientific Pandemic Influenza Group on Modelling (SPI‐M) paper predicted that nearly all care homes would become affected if current conditions persisted and indicated a role for staff in introducing infections, particularly where staff worked across more than one home.^5^ Recent studies indicate that Personal Protective Equipment (PPE) and number of staff employed^6^, ^7^ have an impact on the number of COVID‐19 infections. More recent data from the Care Quality Commission suggest that, to mid‐June, 36% of care homes experienced an outbreak (defined as a single laboratory‐confirmed case)^7^ with a study of care homes across a large Scottish Health Board reporting a figure of 37%.^7^

Early estimates of the impact of COVID‐19 in the UK suggested that inpatient and critical care bed capacity could be overwhelmed.^8^ Hospitals in the UK prepared rapidly for the increase in cases, including cancellation of elective procedures and expediting discharges to home or social care facilities. Testing for residents scheduled for discharge was not always available or done.^9^ Media reports have implicated these discharges as the cause for many of the subsequent outbreaks in care homes^10^, ^11^, ^12^, ^13^ but we were unable to locate any studies either published or in preprint that linked data on discharges to outbreaks. Expert commentary on existing data identified care and non‐care staff, visitors and resident discharges from hospital as possible vectors for the introduction of COVID‐19 into care homes, particularly where testing is not available,^14^ and the discharge back to their care home of untested SARS‐CoV‐2 positive individuals has been suggested as a risk factor for outbreaks in these settings.^15^, ^16^ Studies reporting evidence from testing all staff and residents in specific care homes have suggested high proportions of asymptomatic cases, particularly amongst older residents, than were initially assumed.^7^, ^14^, ^17^ This suggests the risk of importing COVID‐19 into care homes via hospital discharge of untested asymptomatic residents has been underestimated.

Several studies have provided further evidence of factors that may have increased the risk of outbreak in care homes. A large survey carried out by the Office for National Statistics suggested frequent use of agency staff or carers and staff working conditions, including provision of sick pay, influence the risk of an outbreak.^18^ Two studies have used routine data to consider a range of risk factors, including resident need, evidenced by services provided (eg nursing care, dementia care), corporate ownership and pre‐COVID‐19 outbreak history.^7^, ^19^

Wales had its first case of COVID‐19 confirmed on 28 February 2020, and also saw a subsequent rise in cases and outbreaks in care homes. Public Health Wales's (PHW) Communicable Disease Surveillance Centre has been undertaking surveillance for outbreaks in care settings since 2015.

We aimed to use our national surveillance framework to test whether the risk of a COVID‐19 outbreak in the period following a discharge from hospital to a care home was increased compared with other periods, in order to better understand the sources of infection and prevent further incidents.

## 2 METHODS

### 2.1 Study population

The study population was all adults living in residential or nursing care homes in Wales, which has seven health boards and a population of 3 152 879.^20^ We defined a care home as a premises registered with Care Inspectorate Wales (CIW) and recorded as supporting adults. A list of all registered care homes providing personal or nursing care to resident adults was downloaded from the CIW website on 20 May 2020; this contained 1073 adult care homes and included data on care home capacity and nursing care provision. The maximum capacity of all adult care homes during the study period was 25 661 places. Data on dementia services were provided by CIW based on a 2019 review of registration records.

### 2.2 Care home outbreak ascertainment

Data on notifications of COVID‐19 cases and outbreaks were sourced from Tarian, the national health protection case and incident management system managed by PHW. Tarian receives patient‐level data on all PCR positive results for SARS‐CoV‐2 in Welsh residents from the laboratory IT system. All cases with a positive result for SARS‐CoV‐2 from the first recorded case in a resident of Wales on 28 February 2020 to 24 June were extracted from Tarian on 25 June 2020, with additional results to 27 June extracted on 27 July. A total of 15 099 positive results for Welsh residents over this period were identified. Records with postcodes matching those recorded for CIW‐registered care homes were identified and addresses manually matched to ensure only those with care home addresses were included. The date on which the specimen was taken was used as the case date in analysis.

The testing policy for care home cases changed during the time period for this analysis. Initially, testing was offered for up to the three most recently symptomatic individuals in homes which had not already recorded a confirmed case. This was increased to up to 5 symptomatic individuals from 15 April, and to all symptomatic residents from 24 April. Due to likely under‐ascertainment of cases in the earlier part of this period, we defined a COVID‐19 outbreak as one resident testing positive for SARS‐CoV‐2 whilst resident (consistent with Burton et al^7^), or within 14 days of being resident. All testing was performed by health boards in Wales and all samples processed by NHS laboratories. From 02 May, all hospital patients were required to have a negative COVID‐19 test result before being allowed to be discharged back into a care home.

### 2.3 Hospital discharge ascertainment

Data were extracted from the Patient Episode Database for Wales (PEDW), a national database recording all episodes of inpatient activity in NHS Wales hospitals. The extract covered the period 22 February (21 days prior to the first case notified to PHW) to 20 June 2020, the most recent date for which data were available. The extract was made on 4 August 2020 and all discharges relating to postcodes matching those recorded for CIW‐registered care homes were identified and addresses manually matched to ensure only those with care home addresses were included (2218 discharge records). In addition, to capture events for which a postcode was inaccurate or not recorded, a search was conducted using presence of known care home name in the first line of the address. This identified a further 913 discharge events for a total of 3131. Discharge records relating to five care homes across two sites could not be allocated to a specific home as the addresses could not be used to distinguish between distinct facilities on single sites. Records relating to them were therefore excluded from analysis, and the final dataset included 3115 discharges across 1068 care homes, with a combined maximum capacity of 25 384 residents.

### 2.4 Data linkage

Data were linked by care home, matching addresses on the CIW registration record with addresses recorded on individual hospital discharges from PEDW, and on test result records reported on Tarian.

### 2.5 Exposure and outcome

The first notification of a case of COVID‐19 in a care home was made to PHW on 15 March 2020 relating to a specimen collected on 14 March 2020; before this date, all homes were considered at risk. Once homes had a case of COVID‐19 confirmed by laboratory test result, they were excluded from further analysis. This was due to the considerable uncertainty in assigning subsequent cases to a chain of transmission within the home, or to external exposure.

Our outcome was the time (from 22 February) to the first laboratory‐confirmed case of COVID‐19 in each care home. We defined a baseline exposure period following a discharge from hospital as 7 to 21 days post‐discharge. Thus, any first case appearing during this window was recorded as being associated with the discharge event. This window was chosen to approximately account for the potentially incubation and infectious period of an asymptomatic or pre‐symptomatic (and thus untested) discharged resident and for subsequent incubation period of cases caused by onward transmission in the home. As described above, testing pathways for outbreak identification were only routinely available for symptomatic care home residents during the study period. We considered this baseline scenario the most likely to capture an outbreak if caused by a discharge event, but also considered a sensitivity analysis in which all 1‐week, 2‐week and 3‐week windows between 0 and 31 days post‐discharge event were analysed.

### 2.6 Statistical analysis

We used a Cox proportional hazards regression model^21^ to estimate the effect of discharge on the rate at which homes first became affected by COVID‐19. Since we defined the (baseline) exposure period as 7‐21 days post‐discharge, we considered the factor “hospital discharge” as a time‐dependent covariate in the model. Thus, any home could potentially move back and forth between the at‐risk or not at‐risk categories over time. Additional covariates investigated were obtained from CIW: size of home, services available (nursing, specialist care for dementia or learning disabilities) and region (health board). Hazard ratios were calculated for the unadjusted univariable models and for the mutually adjusted full model. In our sensitivity analysis, we considered the wide range of possible exposure windows (between 0 and 31 days), controlling for the false discovery rate using *q* values.^22^ We also calculated outbreak event rates per 1000 days of exposure to hospital discharge compared with the event rate per 1000 days unexposed, and stratified these by care home size.

**FIGURE 1:**
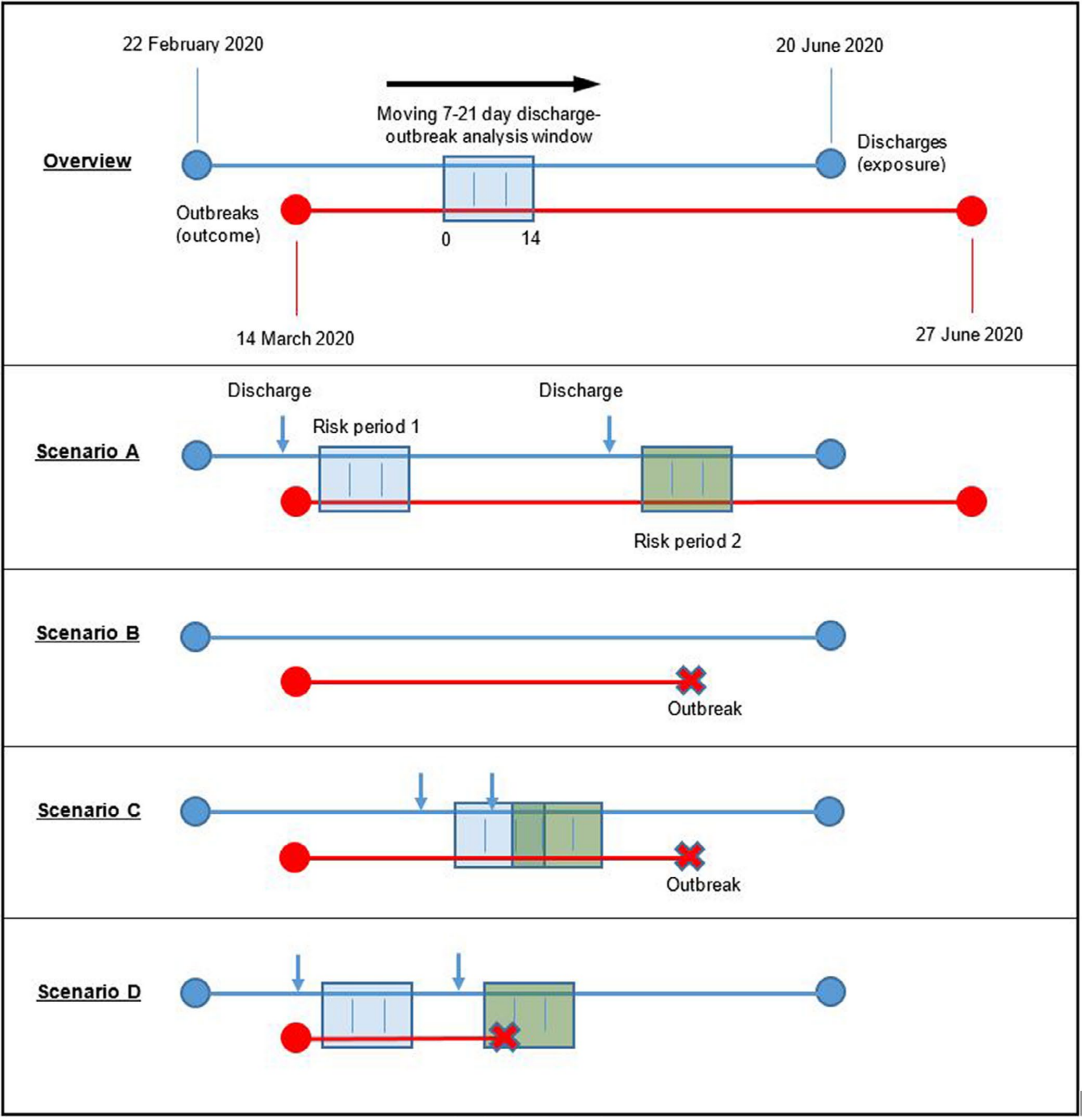
Study period analysis timeline with risk period interaction scenarios. Scenarios: (A) Two non‐overlapping exposure periods, no outbreak; (B) No exposure to hospital discharge, outbreak occurs; (C) Two overlapping periods of exposure, outbreak occurs later when not exposed; (D) Two non‐overlapping periods of exposure, outbreak occurs during the second discharge period

This study period timeline is shown in Figure 1. The overview depicts how the 7‐21 day risk period follows a discharge date. Each time there was a discharge to a home, a new risk period was added to the model. Depending on timing of discharges to a home, risk periods in that home could be consecutive (scenario A), not occur (scenario B) or be overlapping (scenario C). In the case of overlapping risk periods, these were considered cumulative in our model, extending the overall risk period. As such, our model accounted for the possibility of care homes having none, some or all risk periods overlapping. Our end point was time to first outbreak, hence, if an outbreak occurred in a home (scenarios B, C and D), it was censored at that point.

### 2.7 Patient and public involvement

Due to the nature of this study (analysis of routine data) and the imperative to analyse data and report results rapidly to support public health responses to COVID‐19, it was not possible to involve patients and the public in this study.

## 3 RESULTS

Of the 1068 care homes in the analysis, an outbreak was recorded in 330 (30.9%), with a total of 1544 recorded cases. 544 homes accepted a discharge. There were outbreaks in 245 of the 544 care homes with a discharge (45.0%). In these homes, 16 experienced the outbreak prior to any discharge. There were 85 outbreaks in homes with no exposure‐creating discharge (16.2%).

**FIGURE 2:**
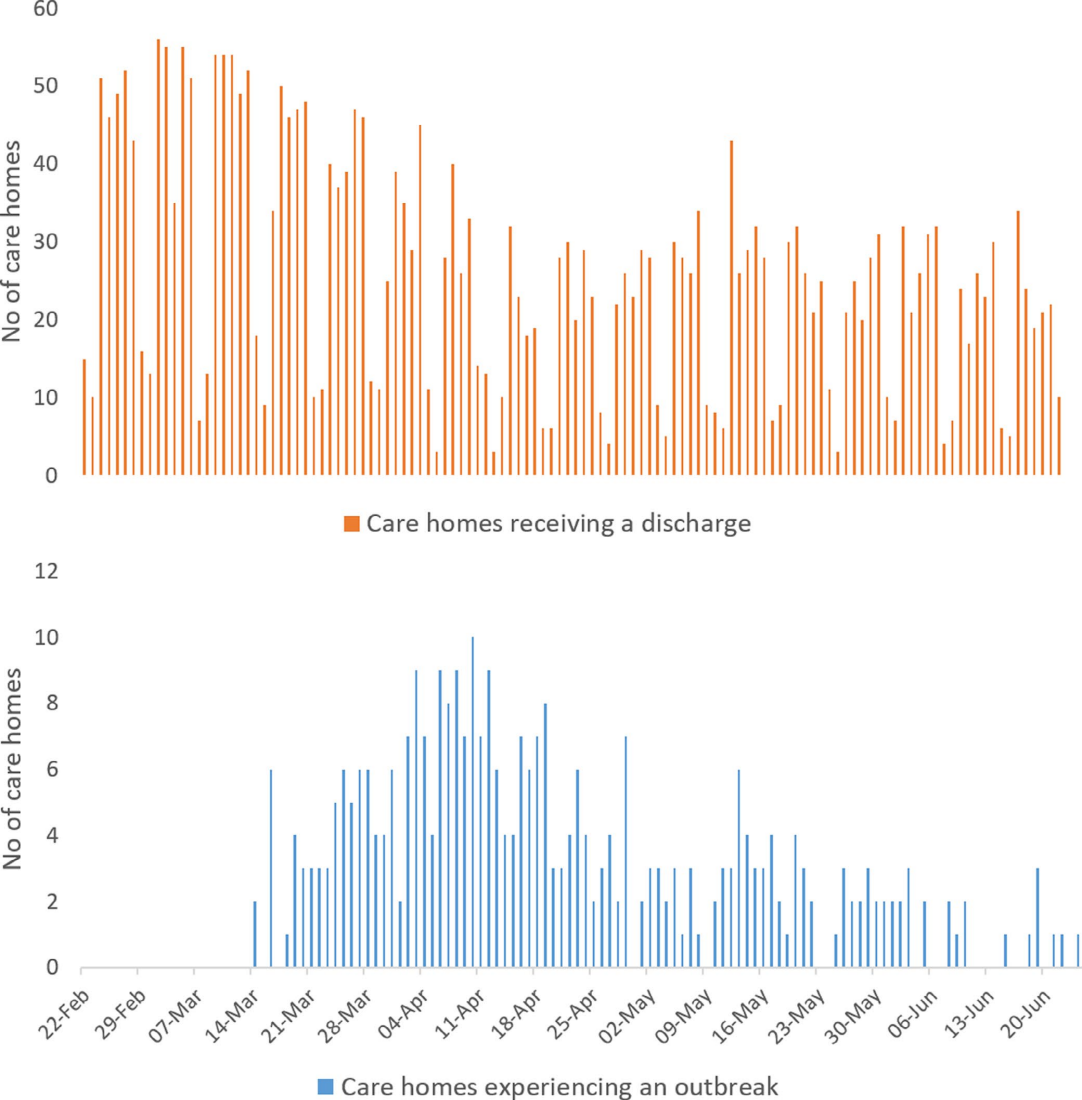
Hospital discharges and outbreaks (first positive SARS‐CoV‐2 tests in resident), care homes in Wales, 22 February to 27 June 2020

Of the 3115 discharges, 1944 were into a care home that reported an outbreak, of which 1058 occurred prior to the outbreak and therefore created or extended a period of exposure. There were 1171 discharges into a care home that did not report an outbreak. Dates for all 330 outbreaks and the 2229 discharges creating or extending an exposure period between 22 February and 27 June 2020 are shown in Figure 2. A summary of the characteristics and hospital discharges of the care homes in the national cohort is given in Table 1.

**TABLE 1:** Summary statistics for hospital discharges and positive SARS‐CoV‐2 tests in residents of care homes in Wales, 22 February to 27 June 2020

Health Board	Total care homes	Total capacity	Total laboratory‐confirmed results	Care homes with outbreak (n)	Care homes with outbreak (%)	Care homes with discharge (n)	Care homes with discharge (%)
Aneurin Bevan	170	4053	233	63	37.1	93	54.7
BCU	290	6730	492	115	39.7	189	65.2
Cardiff & Vale	130	3291	192	44	33.8	55	42.3
CTM	119	2967	208	40	33.6	69	58.0
Hywel Dda	185	3869	175	17	9.2	100	54.1
Powys	40	1227	46	8	20	9	22.5
Swansea Bay	134	3247	198	43	32.1	29	21.6
Wales total	1068	25 384	1544	330	29.4	544	50.9

In the Cox regression, time‐dependent exposure to hospital discharge in the univariable model, with no other factors, was associated with a significantly increased hazard ratio for the risk of an outbreak (2.47, 95% CI: 1.96, 3.11, see Table 2). Similarly, significant univariable effects of size, dementia care, service subtype (nursing care), learning disability provision and regional health board were detected. However, in the mutually adjusted model, there was no significant association for hospital discharge, service subtype, dementia care or learning disability provision. The adjusted hazard ratio for hospital discharge was slightly raised, at 1.15, but with a 95% CI from 0.89 to 1.47 (*P* = .29). The results indicate strong confounding in the raw data by care home size, which was by far the strongest independent predictor of outbreak risk. In comparison with the reference category of small care homes with 1‐9 residents, the hazard ratio for homes with 10‐24 residents was 3.40 (1.99, 5.80). For homes of 25‐49, the hazard ratio was 8.25 (4.93, 13.81) and for the largest category of homes (50+) it was 17.35 (9.65, 31.19). The effect of health board largely mirrored the regional size of the epidemic and therefore acted as a marker of prevalence. Proportional hazard assumptions were met in all models, as assessed by non‐significant global test for time trend in residuals.

**TABLE 2:** Hazard ratios for care home COVID‐19 outbreaks following discharge from hospital (crude and adjusted by size and type and level of care, significant hazard ratios highlighted in red), Wales, 29 February to 27 June. (1) Excludes 19 homes which reported provided care without nursing for adults and children

	Descriptive analysis				Univariable Cox regression			Multivariable Cox Regression		
	Number of Homes	Proportion of all homes (%)	Number of Outbreaks	Proportion of homes with outbreak (%)	Hazard Ratio	Lower 95% CI	Upper 95% CI	Hazard Ratio	Lower 95% CI	Upper 95% CI
Maximum capacity										
Under 10	382	35.8%	25	6.5%	**‐**	‐	‐	‐	‐	‐
10‐24	241	22.6%	54	22.4%	**3.70**	*2.30*	*5.95*	**3.40**	1.99	5.80
25‐49	336	31.5%	165	49.1%	**10.20**	*6.69*	*15.54*	**8.25**	4.93	13.81
50+	109	10.2%	86	78.9%	**22.36**	*14.29*	*35.00*	**17.35**	9.65	31.19
Dementia										
No Dementia Care	437	40.9%	76	17.4%	**‐**	**‐**	**‐**	**‐**	**‐**	
Dementia Care	529	49.5%	240	45.4%	**3.19**	*2.47*	*4.14*	**1.20**	0.90	1.60
Unknown Dementia Care	102	9.6%	14	13.7%	**0.79**	*0.45*	*1.40*	**0.87**	0.49	1.54
Service subtype*(1)*										
Adults without Nursing	791	74.1%	185	23.4%	**‐**	**‐**	**‐**	**‐**	**‐**	‐
Adults with Nursing	258	24.2%	145	56.2%	**3.16**	*2.54*	*3.93*	**0.96**	0.74	1.24
Provision for learning disability										
No Provision	620	58.1%	263	42.4%	**‐**	**‐**	**‐**	**‐**	**‐**	
Provision	448	41.9%	67	15.0%	**0.30**	*0.23*	*0.39*	**0.86**	0.63	1.16
Health Board										
Aneurin Bevan	170	15.9%	63	37.1%	**1.28**	*0.87*	*1.88*	**1.26**	0.85	1.86
Betsi Cadwaladr	290	27.2%	115	39.7%	**1.17**	*0.83*	*1.67*	**1.05**	0.73	1.52
Cardiff & Vale	130	12.2%	44	33.8%	**1.09**	*0.72*	*1.66*	**1.25**	0.82	1.93
Cwm Taf Morgannwg	119	11.1%	40	33.6%	**1.05**	*0.86*	*1.61*	**1.07**	0.69	1.66
Hywel Dda	185	17.3%	17	9.2%	**0.24**	*0.14*	*0.42*	**0.24**	0.13	0.42
Powys	40	3.7%	8	20.0%	**0.55**	*0.26*	*1.18*	**0.40**	0.19	0.86
Swansea Bay	134	12.5%	43	32.1%	**‐**	**‐**	**‐**	**‐**	**‐**	**‐**
Discharge										
No discharge	524	49.1%	85	18.7%	**‐**			**‐**		
Discharge	544	50.9%	245	43.4%	**2.47**	*1.96*	*3.11*	**1.15**	0. 89	1.47
All Homes	1068	100.0%	330	30.9%	‐	*‐*	*‐*	‐	‐	‐

The confounding effect of care home size on observed univariable effect of hospital size can clearly be seen by considering the outbreak event rate per 1000 days at risk from hospital discharge (within the window) and comparing it to the event rate when not exposed. Over all care homes, there was a recorded 6.67 outbreaks per 1000 days of exposure to hospital discharge, compared to 2.47 outbreaks per 1000 days not in the exposed window. However, after stratifying by home size there were no significant differences at any care home size category. For example, the largest (50+) care homes recorded 14.05 (95% CI 10.08, 18.22 per 1000 days when exposed to a hospital discharge, and a similar 11.69 (95% CI 8.53, 14.99) outbreaks per 1000 days when unexposed (see Table 3).

**TABLE 3:** Outbreaks by care home capacity, within and outside of risk period, 22 February to 27 June 2020.

Maximum capacity	Risk period (7‐21 d)	Days in study	Number of outbreaks	Rate of outbreaks per 1000 d	Lower 95% Confidence interval	Upper 95% Confidence interval
Under 10	Not in Risk Period	42 830	23	0.54	0.34	0.76
	Within Risk Period	1148	2	1.74	0.21	5.48
10‐24	Not in Risk Period	21 020	43	2.05	1.48	2.64
	Within Risk Period	4317	11	2.55	1.27	4.22
25‐50	Not in Risk Period	19 716	105	5.33	4.36	6.26
	Within Risk Period	8718	60	6.88	5.25	8.53
Over 50	Not in Risk Period	3850	45	11.69	8.53	14.99
	Within Risk Period	2919	41	14.05	10.08	18.22
All Homes	Not in Risk Period	87 417	216	2.47	2.15	2.76
	Within Risk Period	17 102	114	6.67	5.50	7.79
Total	All	104 519	330	3.16	2.83	3.46

In our sensitivity analysis, considering a wide range of possible time‐dependent exposure windows, no q values for hospital discharge reached significance at either the 5% or 10% level. The estimated overall proportion of true null hypotheses (π_0_), was 1.0. The smallest *q* value was 0.14, associated with an observed hazard ratio of 1.43 at a window of 10 to 31 days, and implying a minimum false discovery rate (fdr) of 14% incurred if considered significant. Very similar results were obtained using the local false discovery rate, and the estimated π_0_ remained 1.0 across all values of fdr tuning parameter *λ*. We note that when considering only hospital discharge and care home size in the model (omitting all other non‐significant covariates) the results were almost identical. Finally, we considered the effect of the change in policy to mandate testing prior to discharge (02/05/20) by fitting the models with a factor for the two time periods. This factor was not found to be significant and did not significantly alter hazard ratios.

## 4 DISCUSSION

Consistent with our study, care home size has been the only factor consistently reported as influencing the risk of outbreak, with Burton et al^7^ describing an odds ratio for outbreak of 3.50 (95%CI 2.06 to 5.94) per 20‐bed increase and Dutey‐Magni et al^19^ finding an adjusted hazard ratio for individual infection of 1.59 (95%CI 2.06 to 5.94) in 45‐59 bed facilities and 1.87 (95%CI 1.44 to 2.43) for 70‐84 beds when compared with 20‐34 bed care homes. It is possible that, because larger homes require more staff and have potentially higher levels of mixing than smaller homes, outbreaks are more likely. These homes are potentially more likely to use agency staff to fill rotas that smaller homes, who might possibly work at more than one care home and present increased opportunities to introduce infection to care homes. Homes serving residents with higher needs would be expected to have higher staff/resident ratios and be less ability to reduce their personal risk of infection through handwashing and minimizing social contacts, which could be a possible reason for increased likelihood of infections in these settings. These structural and operational parameters of care homes are areas which warrant further investigation.

It must be noted that although a large number of care homes and events were included in the analysis, the precision of our estimated hazard ratio for the effect of hospital discharge covers the confidence interval 0.9 to 1.5. Hence, an effect within this range cannot be ruled out, and in individual cases, the source of the introduction to the home could have been hospital discharge. Whilst it is possible that few infectious cases were discharged, or they were late in infection so not excreting, it is also possible that care home staff took specific action receiving discharged patients meaning these residents were successfully isolated in the homes. In addition, the potential increased risk of acquiring COVID‐19 had they not been discharged to care homes should be considered. Remaining in hospital is not without risk, and there was a rationale for expediting discharges, given the expected influx of COVID‐19 cases to hospitals.

### 4.1 Limitations

Clearly, not all discharges would have had COVID‐19, so the effect of our defined risk factor would be diluted by non‐risk discharges. However, the aim was to see an overall effect of the pattern and policy of discharges. It was not possible in this study to link data at an individual level and therefore to ascertain if the case in outbreaks was the resident who had been discharged from hospital within the period of interest. This will be the focus of further investigation. The matching of cases to discharges could be investigated to assess if a case was the primary case discharged from hospital or a secondary infection within the home. Further study will focus on understanding how many homes care home staff worked in during the study period, especially if agency staff were working across multiple homes each week. Here, we focused on the timing of the first outbreak. An analysis of the timeline of all cases is complicated by very limited information on the balance of internal and external exposure, as well as changing testing practices. Such an analysis could shed light on the time‐dependent intensity of cases, and what external factors may have been contributing to that.

## 5 CONCLUSIONS AND RECOMMENDATIONS

The requirement for a hospital patients to provide a negative RT‐PCR for SARS‐CoV‐2 remains in place at the time of writing (December 2020). The Welsh Government's Technical Advisory Group have reviewed testing criteria for those transferring from hospital to care home settings with a recommendation that the current approach continues.^23^ Whilst the number of cases associated with care homes stopped rising following the requirement to test on discharge,^24^ the number of care home associated incidents began to rise again in October, alongside increases in the number of new cases in the community.^25^ This suggests that alternate sources for seeding residential care outbreaks should be investigated, including the risks to and from staff and the overlap with other community transmission.

Larger homes were at considerably greater risk of COVID‐19 outbreaks, but for the period studied, the risk was not significantly increased in the period following a hospital discharge. Further analyses should investigate the risk where discharges were confirmed or probable cases of COVID‐19, and also consider additional evidence on likely chains of transmission that may become available from sources such as greater record linkage and viral genetic sequence data. Patients who are infectious with COVID‐19 or other infections can seed outbreaks into residential care and other settings, so strict policies to test and isolate care home residents on transfer from hospital are very important to avoid outbreaks. Some of the outbreaks documented here may have been due to hospital discharges. However, overall, these discharges were not a significant factor in the spread of COVID‐19 to residential care in Wales.

## CONFLICT OF INTERESTS

None declared.

## AUTHOR CONTRIBUTION

**Chris Emmerson:** Data curation (lead); Formal analysis (equal); Visualization (lead); Writing‐review & editing (lead). **James P. Adamson:** Supervision (equal); Writing‐original draft (lead); Writing‐review & editing (equal). **Drew Turner:** Data curation (equal); Formal analysis (equal). **Mike B. Gravenor:** Formal analysis (lead); Investigation (equal); Methodology (equal); Supervision (equal); Writing‐review & editing (equal). **Jane Salmon:** Writing‐review & editing (equal). **Simon L. Cottrell:** Writing‐review & editing (equal). **Victoria Middleton:** Data curation (equal); Writing‐review & editing (equal). **Buffy Thomas:** Data curation (equal); Writing‐review & editing (equal). **Brendan W. Mason:** Conceptualization (equal); Writing‐review & editing (equal). **Christ J. Williams:** Conceptualization (equal); Formal analysis (equal); Methodology (equal); Supervision (lead); Writing‐review & editing (equal).

## ETHICS

Ethical oversight of the project was provided by PHW R&D Division. As this work was carried out as part of the health protection response to a public health emergency in Wales, using routinely collected surveillance data, PHW R&D Division advised that NHS research ethics approval was not required. The use of named patient data in the investigation of communicable disease outbreaks and surveillance of notifiable disease is permitted under Public Health Wales's Establishment Order. Data were held and processed under Public Health Wales's information governance arrangements, in compliance with the Data Protection Act, Caldicott Principles and Public Health Wales guidance on the release of small numbers. No data identifying protected characteristics of an individual were released outside Public Health Wales.

## DISSEMINATION DECLARATION

Public Health Wales is working closely with Welsh Government and other stakeholders including Care Inspectorate Wales in the response to COVID‐19. The results of this study will be made available to all of these stakeholders through the appropriate channels.

## Data Availability

Deidentified data available on request due to privacy/ethical restrictions. Code used to analyse data was written in R and is available to download at: https://github.com/ChrisEmmerson/Carehome_hospital_discharge_study.
